# Snow viruses and their implications on red snow algal blooms

**DOI:** 10.1128/msystems.00083-24

**Published:** 2024-04-22

**Authors:** Adam R. Barno, Kevin Green, Forest Rohwer, Cynthia B. Silveira

**Affiliations:** 1 Division of Biological and Environmental Science and Engineering, King Abdullah University of Science and Technology, Thuwal, Saudi Arabia; 2 Department of Biology, San Diego State University, San Diego, California, USA; 3 Viral Information Institute, San Diego State University, San Diego, California, USA; 4 Department of Biology, University of Miami, Miami, Florida, USA; University of California Irvine, Irvine, California, USA

**Keywords:** bacteriophage, virus, auxiliary metabolic genes, virulent, temperate

## Abstract

Algal blooms can give snowmelt a red color, reducing snow albedo and creating a runaway effect that accelerates snow melting. The occurrence of red snow is predicted to grow in polar and subpolar regions with increasing global temperatures. We hypothesize that these algal blooms affect virus-bacteria interactions in snow, with potential effects on snowmelt dynamics. A genomic analysis of double-stranded DNA virus communities in red and white snow from the Whistler region of British Columbia, Canada, identified 792 putative viruses infecting bacteria. The most abundant putative snow viruses displayed low genomic similarity with known viruses. We recovered the complete circular genomes of nine putative viruses, two of which were classified as temperate. Putative snow viruses encoded genes involved in energy metabolisms, such as NAD^+^ synthesis and salvage pathways. In model phages, these genes facilitate increased viral particle production and lysis rates. The frequency of temperate phages was positively correlated with microbial abundance in the snow samples. These results suggest the increased frequency of temperate virus-bacteria interactions as microbial densities increase during snowmelt. We propose that this virus-bacteria dynamic may facilitate the red snow algae growth stimulated by bacteria.

**IMPORTANCE:**

Microbial communities in red snow algal blooms contribute to intensifying snowmelt rates. The role of viruses in snow during this environmental shift, however, has yet to be elucidated. Here, we characterize novel viruses extracted from snow viral metagenomes and define the functional capacities of snow viruses in both white and red snow. These results are contextualized using the composition and functions observed in the bacterial communities from the same snow samples. Together, these data demonstrate the energy metabolism performed by viruses and bacteria in a snow algal bloom, as well as expand the overall knowledge of viral genomes in extreme environments.

## INTRODUCTION

As the snow melts in mountain regions of British Columbia, Canada, green algae from the genera *Sanguina*, *Chloromonas*, and *Chainomonas* bloom and produce pigments that change the color of the snow surface to red, orange, and green (1, 2). These algae lower the surface albedo and warm the surrounding snow via increased heat absorption, creating a positive feedback loop of algal growth and melting snow (3–5). Indeed, red snow has been shown to be a significant contributor to snowmelt in glaciers and snow-covered mountains in the Pacific Coast Ranges (6, 7). Red snow algal blooms also accelerate bacterial growth and change the taxonomic and functional structure of associated bacterial communities (8–11). Red snow growth leads to increased photosynthesis, carbon fixation, and methane metabolism from primary producers associated with the snow algae, as well as increased heterotrophic activity and viral lysis (11). In contrast, microbial functional pathways enriched in white snow are related to glycan biosynthesis, with relatively fewer signatures of primary productivity than in red snow (11). Furthermore, bacterial heterotrophy dominates the surface snow of Greenlandic sea ice snow, although primary producers are also found in this region (12).

The bacteria in snow and glacial systems display unique cryophilic features, such as an increased amount of unsaturated fatty acids and carotenoids, which increase cell membrane fluidity and stability at low temperatures (13, 14). Bacteria associated with surface snow also contain a higher proportion of genes related to resistance to photochemical stress and base excision repair, which repairs DNA indirectly damaged by reactive oxygen species (12). Snow bacteria have the highest growth rates during snowmelting and have a positive influence on the growth of snow algae (3, 8, 15–18). In fact, snow algal growth is higher in the presence of snow bacteria when compared to antibiotic-treated snow (9, 19). These observations suggest the possibility of mutualistic relationships between snow algae and associated bacteria (10). However, the bacterial populations in snow may also be influenced by local environmental conditions, as similarities in bacterial operational taxonomic units (OTUs) exist across gradients of snow algae density in mountainous regions (20).

Bacteriophages (phages), viruses that infect bacteria, significantly affect bacterial population dynamics, ecology, and evolution in all aquatic, terrestrial, and animal-associated microbiomes (21). Phage top-down control of bacteria through virulent infection increases rates of organic matter turnover and nutrient remineralization (21). Therefore, virulent infections are the best-understood type of virus-bacteria ecological interactions. Yet, half of all known viruses are temperate, which means they are capable of both lytic/virulent infections and lysogenic infections, where the viral DNA is integrated into their host’s genome as prophages (22). During lysogenic infections, phages and their hosts establish a commensal or mutualistic relationship, with distinct outcomes for host ecology. Lysogeny may increase competitive fitness through protection against further virulent infections and may facilitate gene transfers through transduction (23–25). These observations from other ecosystems lead to the hypothesis that snow viruses also affect microbial dynamics and, potentially, red snow blooms (11, 26).

Phage-encoded auxiliary metabolic genes (AMGs) modify the metabolic pathways of their microbial hosts during infection (27, 28). AMGs can also complement bacterial metabolisms, via the acquisition of alternative energy sources under starvation and UV stress protection (27, 29, 30). Both scenarios are relevant in the snow environment (31).

Very little is understood about the role of viruses on the ecology and genomics of snow microbial communities. Metagenome-assembled genomes and phage genomic fragments recovered from Antarctic snow metagenomes revealed widespread dispersal of bacterial groups (mainly *Ralstonia*) and the phages that infect them (32). Widespread phage dispersal has also been observed in semi-isolated cryoconite holes (indentations in snow formed by inorganic debris buildup) located on glaciers in Svalbard, Greenland, and Alpine regions (33). Furthermore, phage transplantation experiments showed that phages from cryoconite holes may have broad host ranges and are composed of 25% temperate phages (34).

Here, we investigated viral and bacterial ecology and genomics in red and white snow from three locations in British Columbia, Canada. While the samples analyzed here for viral abundance displayed low virus-to-microbe ratios (VMRs) compared to other ecosystems, the dominant putative snow viruses were virulent phages genetically distinct from known viruses. Snow virus genomes encoded AMGs involved in energy production and protection against host defense, which are typically involved in virulent infections. The proportion of temperate phages in the snow samples was positively correlated with microbial abundances. These results suggest that lysis-lysogenic switches take place during red snow bloom and snowmelt, potentially affecting bloom progression.

## MATERIALS AND METHODS

### Sample collection

Snow samples (30 L) were collected in June 2017 in the Whistler region of British Columbia, Canada. Five samples were collected from three different locations using a sterile shovel and 30-L bags: Blackcomb Mountain (BC) on the Catskinner Ski Run (red snow: Blackcomb Mountain red snow [BCRS], 50°5′44.8188″N, 122°54′13.4388″W, elevation = 1,826 m, collected 5 June 2017; white snow: Blackcomb Mountain white snow [BCWS], 50°5′43.0188″N, 122° 54′13.2012″W, elevation = 1,831 m, collected 5 June 2017), adjacent to Callaghan Creek (CA) near the Callaghan Ski Run (red snow: Callaghan Pass red snow [CARS], 50°8′59.19″N, 123°7′18.4188″W, elevation = 829 m, collected 6 June 2017; white snow: Callaghan Pass white snow [CAWS], 50°8′59.1612″N, 123°7′13.8″W, elevation = 847 m, collected 6 June 2017) and on the Sixteen Mile Creek Forest Service Road on Cougar Mountain (red snow: Cougar Mountain red snow [CGRS], 50°10′50.304″N, 122°58′25.0356″W, elevation = 989, collected 7 June 2017). We did not collect taxonomic data on the algae species causing the sampled blooms, which could belong to the genera *Sanguina*, *Chloromonas*, or *Chainomonas* and focused instead on the bloom presence as manifested through red snow color. Within 2 hours of collection, snow samples were melted at room temperature and filtered through a 25-µm Nitex mesh filter to remove large debris.

### Bacterial and viral abundances from the Whistler region

For bacterial abundance determination, 1 mL from the 25-µm filtered snowmelt water samples was fixed with 2% paraformaldehyde. These subsamples were passed through a 0.02-µm Anodisc filter (Whatman, UK), and the particles remaining on the filter were stained with SYBR Gold (Life Technologies, USA), mounted on slides, and analyzed by epifluorescence microscopy (35). Ten random images were obtained using ×600 magnification from each snow sample, and bacteria and free virus-like particles (VLPs) were counted using Image-Pro Plus, according to particle size (Media Cybernetics, Maryland, USA). The total number of free VLPs and bacteria per milliliter of snowmelt water was then calculated using the numbers obtained from the microscopy images corrected for the area of each field of view. VMRs obtained from the snowmelt water samples were compared with published VMRs from 23 studies covering 11 ecosystems (23).

### DNA extraction and sequencing

The remaining 25-µm filtered snowmelt water (~30 L) was concentrated using a 100-kDa tangential flow filtration system to a final volume of 500 mL. The concentrate was filtered through a Sterivex 0.45-µm PVDF membrane filter. Chloroform was added to the filtrate at a final concentration of 0.5% and stored at 4°C until further purification of the viral fraction in the laboratory. The membrane filter was stored at −20°C until DNA extraction.

Viral concentrates were further concentrated in the laboratory using 10% (wt/vol) polyethylene glycol 8000. After overnight incubation, the concentrates were centrifuged at 11,000 × *g* for 40 min at 4°C. Supernatants were removed, and the concentrated pellets were resuspended in 8 mL of saline magnesium (SM) buffer (36), which was then overlayed onto a cesium chloride gradient containing 1 mL each of 0.02-µm filtered cesium chloride-SM buffer solutions at successive densities of 1.2, 1.35, 1.5, and 1.7 g/mL and centrifuged at 82,844 × *g* (22,000 RPM) for 12 hours at 4°C (36). After centrifugation, 1 mL of the 1.5-g/mL fraction containing the enriched viral fraction was extracted. This technique selects for double-stranded DNA bacteriophages, which are the most abundant environmental viruses, although other DNA viruses may be present in the samples after this enrichment step. The purity of the viral fraction was verified by visualizing 100 µL of each sample stained with SYBRGold on an epifluorescence microscope. DNA was extracted from the concentrated viral particles following the formamide/cetyltrimethylammonium bromide and phenol/chloroform methods outlined in references 35, 36. Extracted viral DNA was resuspended in 50 µL of molecular-grade water. Removal of microbial DNA was verified using 16S rDNA PCR with the primers 27F (5′-AGR GTT TGA TCM TGG CTC AG′3′) and 1492R (5′-GGH TAC CTT GTT ACG ACT T-3′). Temperature cycling for the PCR was 95°C for 5 min, followed by 30 cycles of 95°C for 0.5 min, 51°C for 0.5 min, and 72°C for 2 min, with 1 final cycle of 72°C for 10 min. Only samples with no 16S amplification in the PCR were kept for further steps. DNA libraries for shotgun metagenomic sequencing of viral concentrates were prepared using an Accel-NGS 1S Plus DNA Library Kit (Swift Biosciences, USA) and sequenced on an Illumina MiSeq platform (Illumina, USA) using Illumina MiSeq Reagent Kit (v.3) (Illumina). Reads were quality-controlled using Bbduk for adaptor trimming, quality trimming (Phred score > 30), quality filtering (Phred score > 30), k-mer filtering (which removes reads with a 31-mer match to an Illumina spike-in), and entropy filtering (>0.90) (37). Because samples were collected in areas that may have had human interference, including ski areas and near service roads, the reads were aligned to the human reference genome GRCh38 database using Smalt with an 80% identity threshold to remove potential contaminants; however, no sequences were removed in this step (38).

Microbial metagenomic DNA was extracted by inserting the 0.45-µm filters in the PowerSoil DNA Isolation Kit (Qiagen, Germany) bead tubes and following the manufacturer’s instructions. Metagenomic DNA libraries were prepared using KAPA Hyper Plus Kits (Roche, Switzerland), and each sample was sequenced in three different lanes (three technical replicates per Roche sample) on an Illumina HiSeq platform (Illumina).

### Taxonomic and functional assignments of bacterial communities

Raw metagenomic reads from the 0.45-µm filter obtained from the five sites were uploaded to the MG-RAST server (39) and quality-controlled via adaptor trimming, quality trimming (Phred score > 15), and removing any human DNA contamination. Sequences were compared to the M5NR database (40) for taxonomic assignment, requiring a minimum length of 15 amino acids with at least 60% identity and an *E* value less than 10^−5^. Relative abundances were calculated by taking the number of hits per taxon divided by the total number of hits. For the functional annotation, sequences were compared to the SEED database with a minimum length of 15 amino acids with at least 60% identity, and an *E* value less than 10^−5^ (40). The SEED database is used to assign genes to functionally related protein families by protein group (level 1), functional group (level 2), metabolic pathway (level 3), and specific function (level 4) (41).

### *De novo* virome analysis

Viromes from the five Canada snow samples were assembled into contigs using metaSPAdes (42). Contigs from each individually assembled sample were combined and clustered by 98% similarity using CD-HIT (43). Contigs longer than 1 kb were submitted to the Virus Identification by IteRative AnnoTation (VIBRANT, v.1.2.1) phage annotation tool (44). VIBRANT uses hidden Markov models (HMMs) for protein annotations and a “v-score” metric to identify viral sequences (44). Functional annotations of viral contigs were determined via HMM searches against the protein families (Pfam), Kyoto Encyclopedia of Genes and Genomes (KEGG), and virus orthologous groups (VOG) databases within VIBRANT. VIBRANT also identified complete circular phages, temperate phages (viruses capable of both virulent and lysogenic infections, defined by the presence of an integrase gene), and phage-encoded AMGs (44). A non-redundant list of viral contigs recruiting more than one read was generated from mapping results, which resulted in 792 unique contigs making up the snow virome database. Post-QC virome and metagenome FASTQ reads were mapped to this database using Smalt with an 80% identity threshold (38). To define the most prominent members of the viral community in the snow samples, the percent abundance of each viral contig per sample was calculated and sorted to select the 10 most abundant contigs per sample. These were selected for further analysis due to their higher coverage. Because nine contigs were shared among the top 10 of two or more samples, there was a total of 36 unique contigs among the top 10 most abundant viral contigs across all samples.

Phylogenomic analysis of the top 36 viral contigs was performed using the ViPTree server (v.1.9), which produced a viral proteomic tree with closely related reference viral genomes from National Center for Biotechnology Information (NCBI) RefSeq and GenBank (45). All of the open reading frames from each viral genome were identified, followed by an all-versus-all tBLASTx (45, 46). These comparisons resulted in normalized similarity *S*_*G*_ scores, which account for both the number of genes shared and the similarity between these genes and were used to calculate genomic distances (1 – *S*_*G*_) and build a proteomic tree using the BIONJ method (45). The 36 viral contigs were compared with the eight most closely related genomes (from genome similarity *S*_*G*_ scores) for each contig. The tree included 131 dsDNA phages and the 36 viral contigs obtained in this study.

Read recruitment by the most abundant contigs was visualized using the Anvi’o metagenomic workflow in Anvi’o (v.6.2) (47). The correlation between the percent abundance of temperate phages and cell abundance counts was tested using the Spearman correlation in the psych R package (48). Metabolic pathway affiliations of the AMGs were derived from the KEGG pathway database (49). AMG abundance was determined as the sum of the percent abundances of each AMG-containing contig. The six complete circular phages containing at least one AMG were visualized using DNAplotter (50).

## RESULTS

### Microbial abundance and community composition

Viral abundances for the five Canada snow samples ranged from 1.1 × 10^6^ VLPs/mL in BCWS to 2.3 × 10^6^ VLPs/mL in BCRS (Table 1). VMRs were higher in the BC samples (VMR = 6.8 in BCWS and 11 in BCRS) than in the CA samples (3.9 in CAWS, 3.1 in CARS) and CGRS (2.1) (Fig. S1). The VMRs found in our snow samples were similar to those found in the open ocean, coastal/estuaries, Antarctic lakes, and sediments (51).

**TABLE 1:** Microbial cell abundances, viral abundances, and virus-to-microbe ratios in the Canada snow samples

Sample	Snow type	Cells (10^6^/mL)	Virus-like particles (10^6^/mL)	Virus-to-microbe ratio (VMR)
BCWS	White	1.6	10.9	6.8
BCRS	Red	2.1	23.2	11
CAWS	White	5.0	19.5	3.9
CARS	Red	4.9	15.2	3.1
CGRS	Red	6.7	14.1	2.1

The most abundant bacterial phyla in the Canada snow metagenomes were Proteobacteria (56% of the total bacterial reads), Bacteroidetes (30%), Actinobacteria (7.2%), Acidobacteria (2.2%), Firmicutes (1.9%), and Cyanobacteria (1.1%) (Fig. S2A). The most abundant bacterial classes were Betaproteobacteria (28% of the total bacterial reads), Alphaproteobacteria (14%), Sphingobacteria (14%), Gammaproteobacteria (12%), and Actinobacteria (7.2%) (Fig. S2B). Metagenomic functional profiles were assigned by classifying reads according to the SEED subsystems levels 1, 2, and 3 (Fig. S3A through C, respectively). Among the eight most abundant SEED level two subsystems were functions related to protein biosynthesis, central carbohydrate metabolism, monosaccharides, lysine, threonine, methionine, and cysteine, and folate and pterins (Fig. S3B).

### Abundant and divergent snow viruses

**Fig 1:**
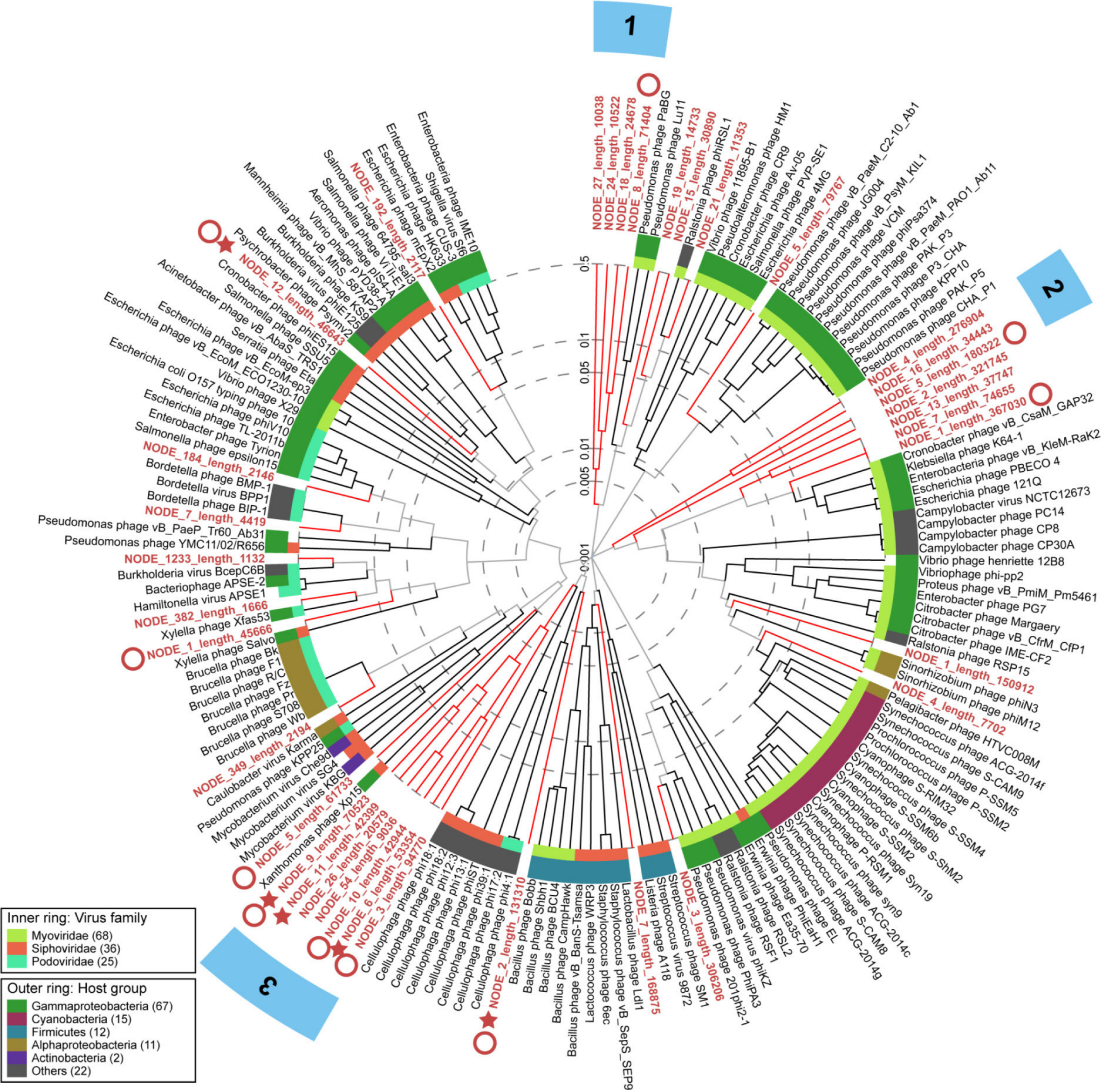
Viral proteomic tree of the 10 most abundant viral contigs and their most related reference genomes. The viral proteomic tree was generated using viral genome sequences and used normalized pairwise tBLASTx scores to calculate genome-wide sequence similarities (represented as branch lengths). The host groups of the reference viruses indicate probable hosts for the newly defined snow viruses. Stars indicate temperate phages from this study. Circles represent putative circular phages. Numbered blue bars denote snow virus clades. Branch lengths are shown in log scale.

**Fig 2:**
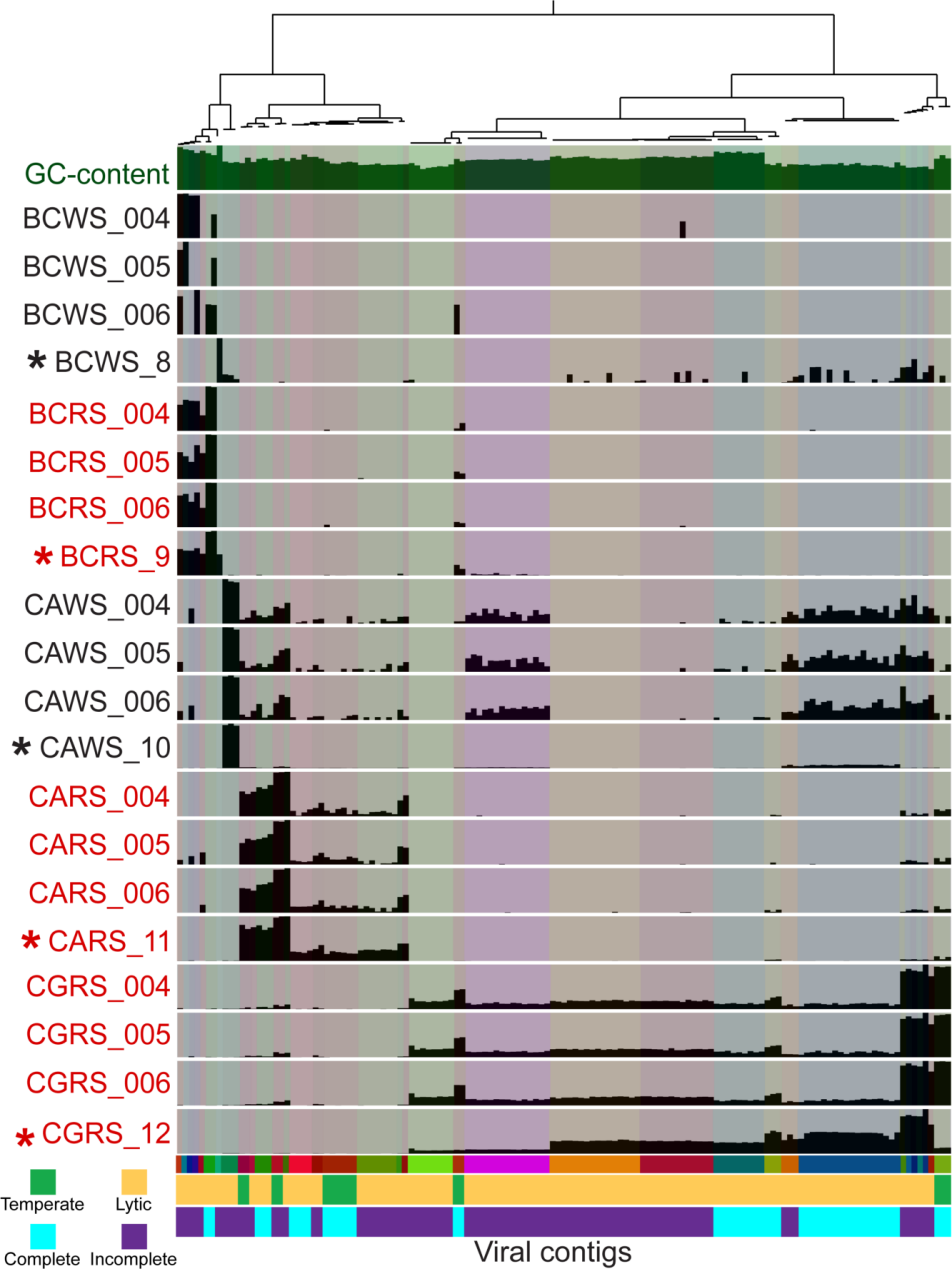
Abundances of the 10 most abundant viral contigs in each of the samples (36 across all samples). Abundances were visualized using the Anvi’o metagenomic workflow. Hierarchical clustering of the contig splits (approximately 20-kb segments of each contig) is done based on Ward’s method using Euclidean distances. The heights of the bars display the mean coverage of each 20 kb of each contig (separated by background column color) divided by mean coverage of all of the contigs. Black labels represent white snow samples, and red labels represent red snow samples. Asterisks denote virome samples. BCWS, Blackcomb Mountain white snow; BCRS, Blackcomb Mountain red snow; CAWS, Callaghan Pass white snow; CARS, Callaghan Pass red snow; CGRS, Cougar Mountain red snow.

Thirty-six unique viral contigs comprised the ten most abundant viral contigs in each sample. Although no viral contig was within the top 10 most abundant in all viromes, nine viral contigs were within the most abundant in two or more samples. One viral contig was shared between the white and red snow from Blackcomb (NODE_349_length_2194); one viral contig was shared between the white snow from Blackcomb and the red snow from Cougar Mountain (NODE_5_length_180322); one viral contig was shared between the red snow from Blackcomb and the white snow from Callaghan Pass (NODE_3_length_306206); five contigs were shared in the white snow from Blackcomb, the white snow from Callaghan Pass and the red snow from Cougar Mountain (NODE_15_length_30890, NODE_18_length_24678, NODE_2_length_321745, NODE_4_length_276904, and NODE_1_length_367030); and one contig was among the top ten in both the white snow from Callaghan Pass and the red snow from Cougar Mountain (NODE_19_length_14733). All other top 10 most abundant viral contigs were unique to their environment. Five of the 36 contigs were classified as temperate phages, while the other 31 were classified as virulent phages based on the presence or absence of VOG-annotated integrase genes (44). The relationship between these 36 contigs and reference viral genomes from the GenomeNet Virus-Host DB (which contains viruses with complete genomes from NCBI RefSeq and GenBank whose accession numbers are listed in EBI Genomes) was visualized using a viral proteomic tree (45) (Fig. 1). The hosts of the reference viruses with the closest relationship with snow viruses were Gammaproteobacteria, Cyanobacteria, Firmicutes, Alphaproteobacteria, and Actinobacteria, reflecting the bacterial community composition in snow. Three snow virus clades with deep branching in this tree were formed (node depth below 0.005), indicating that these contigs represent novel viral taxa from the snow biome with low genetic similarity with known viruses (46). Clade 1 was most closely related to two *Pseudomonas* phages, one *Ralstonia* phage, and three other snow viruses from this study. The most closely related reference genomes to clade 2 were a *Cronobacter* phage, a *Klebsiella* phage, an *Enterobacter* phage, two *Escherichia* phages, and four snow viruses from this study. Clade 3 formed a group of distantly related viruses containing both virulent and temperate phages whose closest relatives were *Cellulophaga* phages. The read recruitment by the snow virome database demonstrated that most contigs were abundant in only one or a few samples (Fig. 2). Red snow recruited reads at a more similar level between samples than the white snow metagenomes and viromes. Nine of the 36 viral contigs were determined to be putative complete circular genomes (44). Two of the nine complete viral genomes were classified as temperate by the presence of an integrase (44).

### Prevalence of temperate phages

Of the 792 unique viral contigs, 20 were classified as being from a temperate phage (2.5%). The relative abundance of temperate phages in the microbial fraction, determined by read recruitment, ranged from 0%–31%, and the average percent abundance of temperate phages across all samples was 11% (Table 2). The relative abundance of temperate phages in the microbial fraction was significantly correlated with cell abundance [Spearman, *r_s_*(13) = 0.66, *P* = 0.008]. In the two sample sites with paired white and red snow samples (BC and CA), the percent abundances of temperate phages were higher in the red snow samples (Table 2).

**TABLE 2:** Abundance of temperate phages at each sample site^*a*^

Sample	Temperate (%)	Virulent (%)
BCWS_L004	0	100.0
BCWS_L005	0	100.0
BCWS_L006	0.391	99.6
BCWS_S8*	1.31	98.7
BCRS_L004	0.967	99.0
BCRS_L005	0.782	99.2
BCRS_L006	1.01	99.0
BCRS_S9*	1.91	98.1
CAWS_L004	11.5	88.5
CAWS_L005	12.0	88.0
CAWS_L006	11.4	88.6
CAWS_S10*	22.3	77.7
CARS_L004	23.3	76.7
CARS_L005	24.5	75.5
CARS_L006	30.6	69.4
CARS_S11*	30.4	69.6
CGRS_L004	22.5	77.5
CGRS_L005	1.3	98.7
CGRS_L006	22.3	77.7
CGRS_S12*	1.16	98.8
Total	11.0	89.0

^*a*^ BCWS, Blackcomb Mountain white snow; BCRS, Blackcomb Mountain red snow; CAWS, Callaghan Pass white snow; CARS, Callaghan Pass red snow; CGRS, Cougar Mountain red snow. Asterisks denote virome samples.

### Auxiliary metabolic genes

In total, the viral contigs encoded 46 unique AMGs, 31 of which were in at least 1 of the top 36 most abundant viral contigs. Only four unique AMGs were found in viruses classified as temperate. The most abundant AMGs across all samples were nicotinamide phosphoribosyltransferase (*NAMPT*), bifunctional NMN adenylyltransferase/nudix hydrolase (*nadM*), dihydrofolate reductase (*folA*), HTH-type transcriptional regulator (*nadR*), and DNA (cytosine-5)-methyltransferase 1 (*DNMT1*).

The AMGs encoded by temperate viruses were *mec* ([CysO sulfur-carrier protein]-S-L-cysteine hydrolase), DNA (cytosine-5)-methyltransferase 3A (*DNMT3A*), *moeB* (molybdopterin-synthase adenylyltransferase), and *sacA* (beta-fructofuranosidase), which are broadly involved in the cysteine biosynthesis (*mec*), DNA methylation (*DNMT3A*), carbohydrate metabolism (*sacA*), and the sulfur relay system (*moeB*).

**Fig 3:**
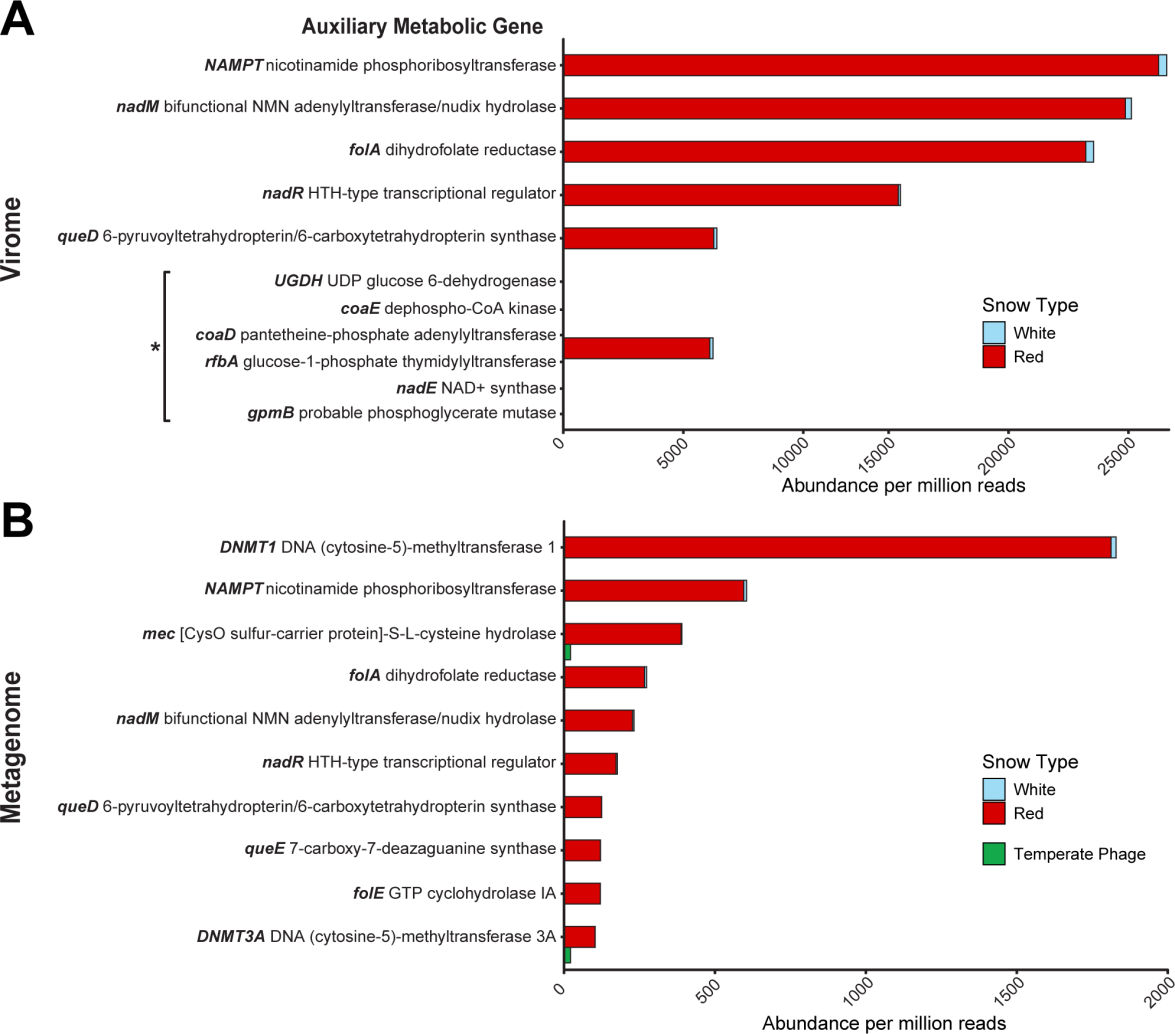
Abundance of dominant AMGs in the virome (**A**) and in viral sequences identified in the metagenome (**B**). Data are shown as the abundance of the AMG-containing contig per one million reads. Five of the most abundant AMGs were shared between the virome and the metagenome. AMGs in temperate phages were found only within the top 10 AMGs in the metagenomic fraction. All the six AMGs indicated by the asterisk were predicted to be encoded by a single putative viral genome fragment (contig NODE_2_length_321745). AMG, auxiliary metabolic gene.

Figure 3A and B show the 10 most abundant AMGs in the viromes and metagenomes, respectively, as determined via read recruitment by the AMG-containing contigs. Five AMGs were shared between virome and metagenome contigs: *NAMPT*, *nadM*, *folA*, *nadR*, and *queD* (6-pyruvoyltetrahydropterin/6-carboxytetrahydropterin synthase). Among the most abundant AMGs that were not shared, the viral fraction had genes related to coenzyme A biosynthesis (*coaE*; dephospho-CoA kinase, and *coaD*; pantetheine-phosphate adenylyltransferase) and NAD^+^ synthesis (*nadE*, NAD^+^ synthase), as well as UDP glucose 6-dehydrogenase, *rfbA* (glucose-1-phosphate thymidylyltransferase), and *gpmB* (probable phosphoglycerate mutase). The uniquely abundant AMGs in the microbial fraction were *DNMT1* and *DNMT3A*, *mec*, *queE* (7-carboxy-7-deazaguanine synthase), and *folE* (GTP cyclohydrolase IA). Two of these AMGs (*mec* and *DNMT3A*) were found in temperate phages, as well as in virulent phages.

**Fig 4:**
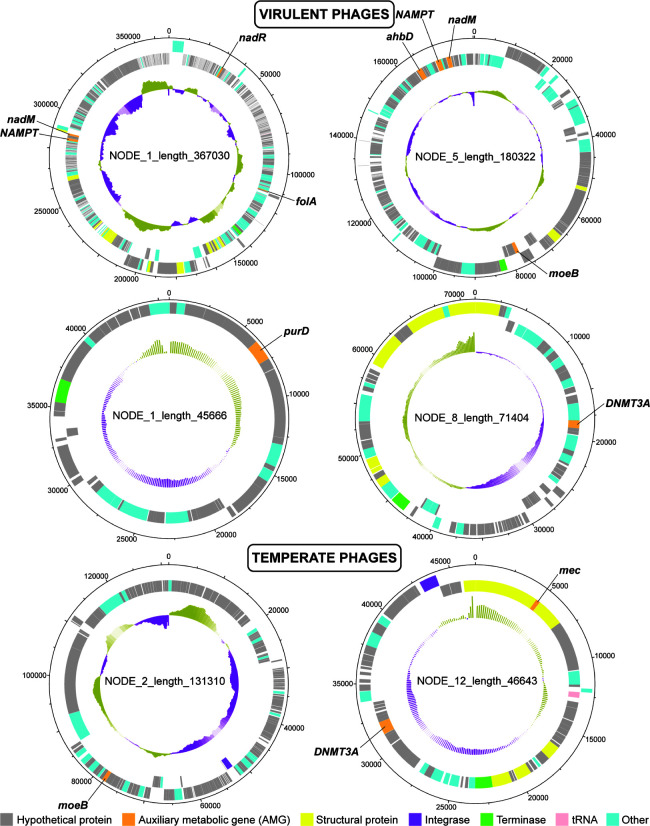
Six complete viral genomes encoding AMGs in snow. Four of the phages were predicted to be virulent, and two (bottom) were predicted to be temperate. The outer ring indicates the nucleotide position in the genome. The two colored middle rings show the predicted genes on the forward strand (outer ring) or reverse strand (inner ring). The innermost ring indicates the percent GC content of the phage genome (higher GC content denoted by the yellow portion, lower GC content denoted by the purple portion). AMGs are annotated. The circular genomes were visualized using DNAplotter (50). AMG, auxiliary metabolic gene.

Six of the nine complete viral genomes encoded the AMGs *NAMPT*, *nadM*, *nadR*, *folA*, *moeB*, *DNMT3A*, and *mec*, which are defined above, as well as *ahbD* (heme synthase), and *purD* (phosphoribosylamine-glycine ligase), which is involved in purine metabolism (Fig. 4). Three of these complete phages contained multiple AMGs (two virulent phages: contigs NODE_1_length_367030 and NODE_5_length_180322, and one temperate phage: contig NODE_12_length_46643).

## DISCUSSION

The effects of phage-bacteria interactions on microbial communities subject to extreme cold, desiccation, and UV exposure in snow environments are relatively unknown (15). This represents a significant knowledge gap considering the increase in global temperatures and expanding red snow events. Here, we show that snow dsDNA viruses are mostly uncharacterized, forming clades of previously unidentified tailed phages. All closely related viruses are tailed phages, which suggests that tailed phages likely also dominate the snow virome. The most abundant novel snow phages have predicted hosts within Gammaproteobacteria.

The most abundant AMGs found in the Canada snow viral genomes indicate that the viruses direct the energy of the host to produce new viral progeny and evade host defenses during virulent infection. Three genes involved in the NAD^+^ salvage pathway from nicotinamide (*NAMPT*, *nadM*, and *nadR*) were among the most abundant AMGs in both the virome and metagenome fractions. *NAMPT* is the rate-limiting step in NAD^+^ synthesis (52), and *nadM* and *nadR* are both adenylyltransferases involved in the NAD^+^ synthesis/salvage pathways (53). Six viral contigs encoded both enzymes (*NAMPT* and *nadM*) necessary to produce NAD^+^ from nicotinamide. Each of these contigs was predicted to be from a virulent phage, including the two complete circular contigs containing both genes (NODE_1_length_367030 and NODE_5_length_180322). Another gene involved in this pathway, *nadE*, was found among the most abundant AMGs in the virome fraction. *nadE* encodes an enzyme involved with synthesizing NAD^+^ *de novo* via different intermediates, a process first discovered in *Francisella tularensis* (54). The production of NAD^+^ salvage enzymes in *Vibrio* phage KVP40 increases upon infection of its host, showing that NAD^+^ biosynthesis after phage infection aids in the production of viral progeny (55). In other ecosystems, NAD^+^-dependent catabolic pathways are more abundant in low-density microbial communities where virulent infection is dominant (51, 56). This suggests that, in snow, phage-encoded AMGs for NAD^+^ metabolism contribute to virulent phage production.

Viral AMG functions are associated with the functional capacities of the microbial hosts. For example, the most abundant SEED level 3 subsystem in the metagenome, respiratory complex I (NADH:ubiquinone oxidoreductase), uses the energy released by the electron transfer from NADH to quinone to pump protons across the plasma membrane (57). The bioavailability of this complex could be modified via the expression of viral auxiliary metabolic genes involved in NAD^+^ biosynthesis. The ubiquitous presence of *nadM* in the snow viromes and the four AMGs involved in NAD^+^ biosynthesis pathways (*NAMPT*, *nadM*, *nadR*, and *nadE*) supports this hypothesis. These genes were found in virulent and temperate phages from red and white snow and indicate how host metabolisms can be modified by infecting phages to control the catabolism in their bacterial hosts.

Dihydrofolate reductase (*folA*) was another abundant gene that functions as a structural protein in phage baseplates (58) and as a metabolic enzyme involved in thymine synthesis (59). The latter function is essential in maintaining the high rate of DNA synthesis that occurs in T4 phage-infected cells (59). Similarly, two genes related to the synthesis of coenzyme A (CoA), *coaD* and *coaE*, were abundant in the virome fractions of the snow samples. CoA is a cofactor that participates in multiple energy metabolism pathways (60). Intermediate metabolites in the coenzyme A biosynthesis pathway have been shown to increase during virulent phage infection (61), although the purpose of this pathway in phage lysis is not entirely known.

The AMGs *queD* (in both the virome and metagenome) as well as *queE* and *folE* (among the most abundant AMGs in the metagenome) encode enzymes involved in the synthesis of 7-cyano-7-deazaguanine from GTP in the queuosine biosynthesis pathway (62–64). Queuosine is a modified base in the wobble position of tRNAs that increases the fidelity of protein synthesis (65). Moreover, 7-deazaguanine derivatives produced in this pathway protect phage DNA from host restriction enzymes without affecting DNA polymerase activity (62, 66). Each of these genes was only found within virulent phages in the Canada snow samples. In a related mechanism, DNA methyltransferases also help phages to avoid host defenses. Two different DNA methyltransferase genes (*DNMT1* and *DNMT3A*) were abundant in the snow viral contigs. Depending on the target sequence, DNA methyltransferases control the expression of genes by adding methyl groups to CpG structures in DNA (67). Canonically, bacteria defend against phage infection via restriction-modification systems consisting of restriction enzymes and methyltransferases (68). By methylating their DNA at specific sites, bacteria distinguish between self-DNA (methylated) and invading phage DNA (non-methylated) and subsequently digest the invading DNA by restriction enzymes. However, certain phages avoid bacterial restriction enzymes by methylating their own DNA during virulent production or prophage induction, thereby disguising the phage DNA within the bacterial host (69–72). DNA methyltransferases were present in both virulent and temperate phages from the Canada snow samples. The abundance of these defense-counter-defense genes suggests that a virulent arms race occurs in snow environments.

While virulent infection appears to be the dominant viral behavior in snow environments, the combination of genomic and density data indicates changes in virus-bacteria interactions in melting snow affected by algae blooms. This could be due to increased microbial growth rates in red snow, fueled by a positive feedback loop of microbial and algal growth as the algae release organic carbon that fuels heterotrophic bacteria (11). High microbial density increases the frequency of phage-host encounters and, therefore, the frequency of lysogeny caused by phage coinfections (51, 73). High densities fueled by the increase in labile organic carbon are also typically associated with metabolic switches toward low-efficiency metabolisms that rely more on NADPH than NADH (74). Therefore, the increase in bacterial densities in red snow leads to the hypothesis that snow communities switch from lysis to lysogeny during red snow blooms, where there is an increase microbial activity and growth (11). The positive correlation between cell abundance and temperate phage relative abundance in the metagenomes of the Whistler region supports this hypothesis [Spearman, *r_s_* (13) = 0.66, *P* = 0.008].

The range of VMRs found in the Canada snow samples (2.1–11.0) was in the same order of magnitude as VMRs observed in snow samples from the European Alps that displayed a median VMR of 1.24 (14). The highest VMRs were found in BCWS and BCRS, with the lowest relative abundances of temperate phages and cell abundances (Tables 1 and 2). Similarly, Fillinger et al. (14) found that VMRs were negatively correlated with prokaryotic cell numbers in three different snow sites in the European Alps across 2 years. Interestingly, nutrient enrichment in the supraglacial meltwater led to an increase in bacterial abundance, correlating with a decrease in VMRs (from nearly 15 to between 1 and 4 over the course of 12 days) (75). These results suggest that density-dependent transitions from virulent to temperate at high microbial abundances, as predicted by the piggyback-the-winner dynamics, also occur in snow ecosystems. Future studies with broader geographical ranges and sample sizes are necessary to understand the biogeographical patterns associated with microbial and viral density and infection dynamics in snow.

### Conclusion

Viruses play essential roles in biogeochemical cycling and microbial ecology in all the studied planet’s biomes. Here, we showed that viruses are key members of the snow microbial community, with potential implications for snowmelt dynamics. Our data indicate that viruses control bacterial populations through lytic induction and redirect the host’s energetic metabolism, likely impacting organic matter turnover. The data also suggest that the increase in microbial abundances in red snow blooms is associated with increasing temperate viral behavior. This trend is predicted to decrease viral top-down control on bacteria, accelerating the bacteria-algae feedback loop.

## Data Availability

Metagenomic sequences are available in the MG-RAST database under the project name “canada_snow_metagenomes.” Raw virome sequences are available in the National Center for Biotechnology Information Short Read Archive under BioProject ID PRJNA819850.

## SUPPLEMENTAL MATERIAL

Supplemental Figures msystems.00083-24-s0001.docx Figures S1 to S3.

ASM does not own the copyrights to Supplemental Material that may be linked to, or accessed through, an article. The authors have granted ASM a non-exclusive, world-wide license to publish the Supplemental Material files. Please contact the corresponding author directly for reuse.
