# Supplementary material for: Snow viruses and their implications on red snow algal blooms
Source: mSystems. 2024 Apr 22;9(5):e00083-24. doi: 10.1128/msystems.00083-24 (PMC11097641; doi:10.1128/msystems.00083-24)
Supplement: Supplemental Figures — Figures S1 to S3. [file msystems.00083-24-s0001.docx]

**Supplementary Figures:**


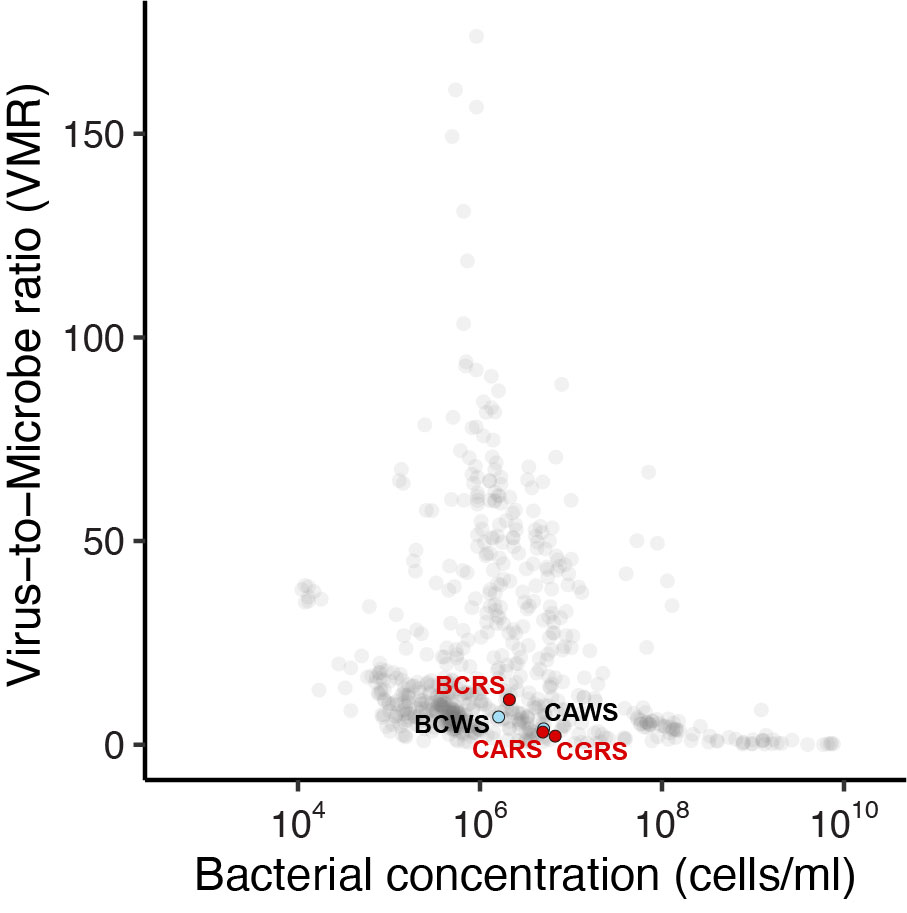


Supplementary Figure 1. Virus-to-microbe ratios (VMRs) of the five snow samples plotted with VMRs from marine, freshwater, soil, and animal-associated ecosystems (from Knowles et al., 2016). Cell abundances, virus counts, and VMR data from the snow samples are in Table 1. BCWS = Blackcomb Mountain White Snow, BCRS = Blackcomb Mountain Red Snow, CAWS = Callaghan Pass White Snow, CARS = Callaghan Pass Red Snow, CGRS = Cougar Mountain Red Snow.


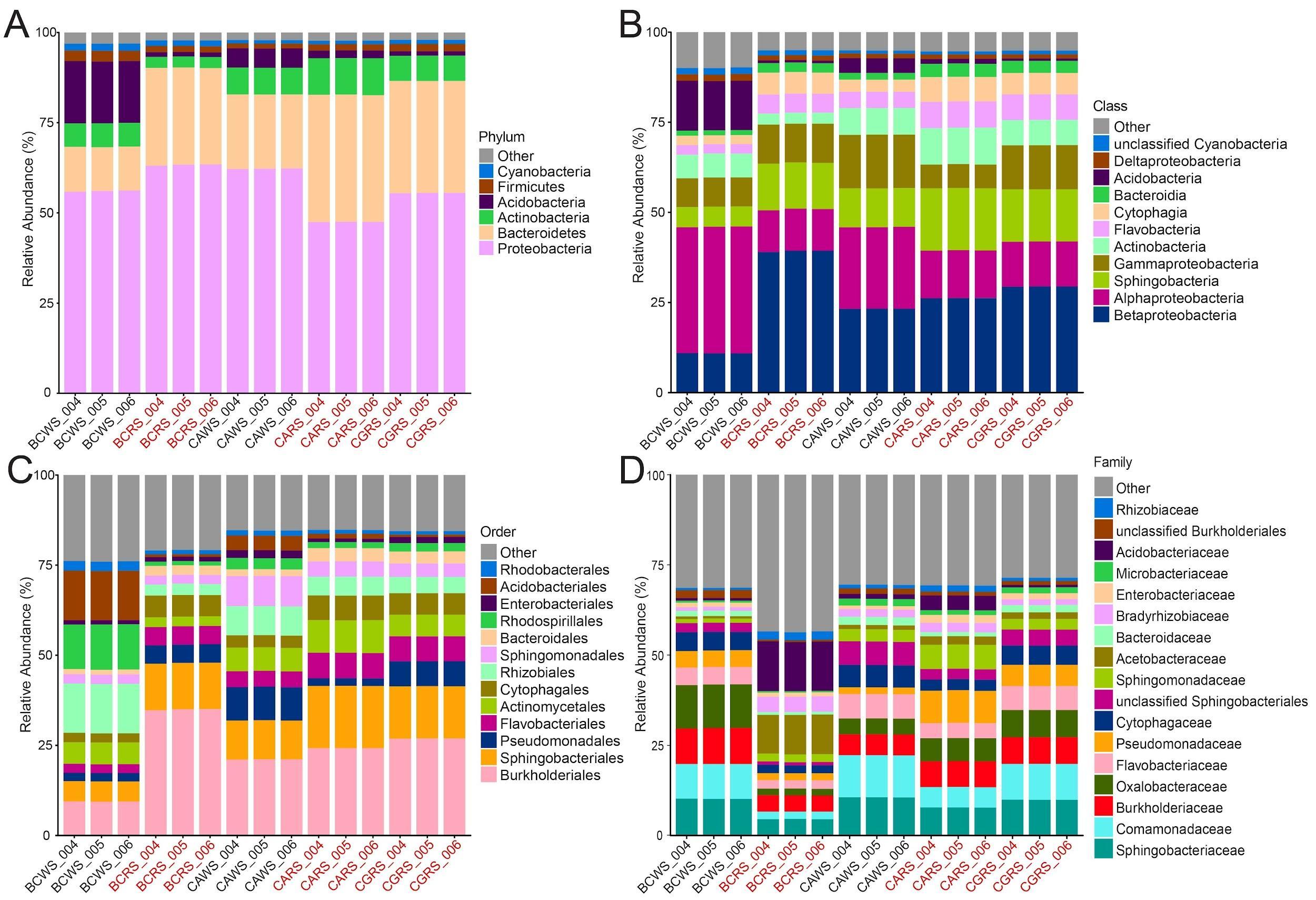


Supplementary Figure 2. Bacterial taxonomic profiles. The plots display taxa comprising over 1% of the total bacterial reads. (A) phylum, (B) class, (C) order, and (D) family. BCWS = Blackcomb Mountain White Snow, BCRS = Blackcomb Mountain Red Snow, CAWS = Callaghan Pass White Snow, CARS = Callaghan Pass Red Snow, CGRS = Cougar Mountain Red Snow.


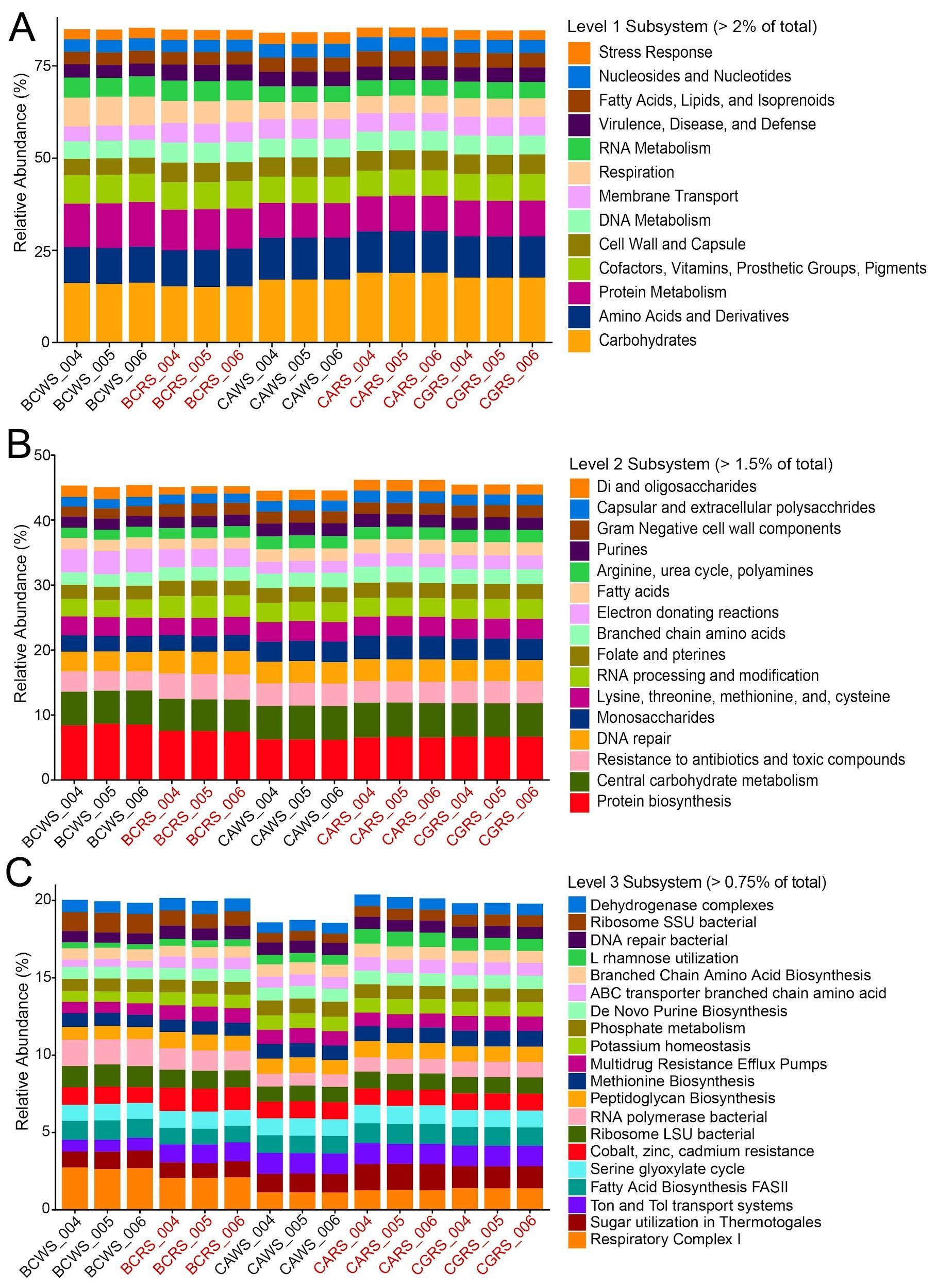


Supplementary Figure 3. The SEED level subsystems for the Canada snow metagenomes. (A) SEED level 1, (B) SEED level 2, and (C) SEED level 3. BCWS = Blackcomb Mountain White Snow, BCRS = Blackcomb Mountain Red Snow, CAWS = Callaghan Pass White Snow, CARS = Callaghan Pass Red Snow, CGRS = Cougar Mountain Red Snow.
